# Response to Tsuda et al. “demonstrating the undermining of science and health policy after the Fukushima nuclear accident by applying the toolkit for detecting misused epidemiological methods”

**DOI:** 10.1186/s12940-023-00966-z

**Published:** 2023-02-17

**Authors:** Enora Cléro, Claire Demoury, Bernd Grosche, Liudmila Liutsko, Yvon Motreff, Takashi Ohba, Deborah Oughton, Philippe Pirard, Agnès Rogel, Thierry Schneider, An Van Nieuwenhuyse, Dominique Laurier, Elisabeth Cardis

**Affiliations:** 1 Health and Environment Division, Institute for Radiological Protection and Nuclear Safety (IRSN), Fontenay-aux-Roses, France; 2 Risk and Health Impact Assessment Unit, Sciensano, Brussels, Belgium; 3 Retired Federal Office for Radiation Protection (BfS), Munich, Germany; 4 Institute for Global Health (ISGlobal), Barcelona, Spain; 5 Pompeu Fabra University (UPF), Barcelona, Spain; 6 Spanish Consortium for Research and Public Health (CIBERESP), Instituto de Salud Carlos III, Madrid, Spain; 7 Unitat de Suport a la Recerca Metropolitana Nord, IDIAP Jordi Gol, Barcelona, Spain; 8 Santé publique France, F-94415 Saint-Maurice, France; 9 Fukushima Medical University, 1 Hikarigaoka, Fukushima, Fukushima 9601295 Japan; 10 Norwegian University of Life Sciences (NMBU), Center for Environmental Radioactivity (CERAD), Aas, Norway; 11 Nuclear Protection Evaluation Centre (CEPN), Fontenay-aux-Roses, France; 12 Centre for Environment and Health, Department of Public Health and Primary Care, Leuven, Belgium; 13 Department of Health Protection, Laboratoire National de Santé (LNS), Dudelange, Luxembourg

**Keywords:** Epidemiological methods, Thyroid cancer, Screening, Overdiagnosis, Nuclear accident

## Abstract

**Background:**

The SHAMISEN (Nuclear Emergency Situations - Improvement of Medical And Health Surveillance) European project was conducted in 2015-2017 to review the lessons learned from the experience of past nuclear accidents and develop recommendations for preparedness and health surveillance of populations affected by a nuclear accident. Using a toolkit approach, Tsuda et al. recently published a critical review of the article by Cléro et al. derived from the SHAMISEN project on thyroid cancer screening after nuclear accident.

**Main body:**

We address the main points of criticism of our publication on the SHAMISEN European project.

**Conclusion:**

We disagree with some of the arguments and criticisms mentioned by Tsuda et al. We continue to support the conclusions and recommendations of the SHAMISEN consortium, including the recommendation not to launch a mass thyroid cancer screening after a nuclear accident, but rather to make it available (with appropriate information counselling) to those who request it.

## Background

The article by Tsuda et al. [1] presents an interesting approach: using a published toolkit to detect the misuse of epidemiological methods. Unfortunately, this article demonstrates that such an approach does not prevent misinterpretation of scientific publications.

This article presents an accumulation of minor points of criticism, unfounded accusations and misinterpretation of our messages. It is not our intention to respond to each of these points, but rather to address the main points of criticism of the article by Cléro et al. [2] concerning the SHAMISEN (Nuclear Emergency Situations - Improvement of Medical And Health Surveillance) European project [3], in order to illustrate the erroneous and biased nature of these criticisms.

## Response to criticism on SHAMISEN

First, Tsuda et al. [1] state the SHAMISEN consortium [2] misused epidemiology by deliberately not citing certain papers. The aim of the SHAMISEN project was to provide evidence-based recommendations to help improve health conditions of affected populations by addressing not only radiation protection and physical health, but also mental health, living conditions after a nuclear accident, communication, and ethical issues [3]. Therefore, the SHAMISEN paper [2] did not aim to be a systematic review of all papers published on the Chernobyl and Fukushima accidents, but aimed to present the lessons learned from thyroid cancer screening. Thus, only the most relevant articles were cited by the authors. We consider that the SHAMISEN paper [2] provides a good overview of the lessons learned from thyroid cancer screening after nuclear accident. This overview was presented to and discussed with a broad set of experts in this domain, notably during a workshop towards the end of the project.

Secondly, Tsuda et al. [1] claim the SHAMISEN consortium [2] failed to point out that most of the publications showing overdiagnosis of thyroid cancer after screening concern unexposed adults and not children. However, evidence suggests that overdiagnosis is similar in children and adults [4]. Vaccarella et al. [4] concluded that “*the pattern of thyroid cancer incidence in children and adolescents mirrors the pattern seen in adults, suggesting a major role for overdiagnosis*” in several countries of the world. We reaffirm the SHAMISEN conclusion that thyroid cancer screening among children and adolescents leads to overdiagnosis [2].

With regard to the authors’ declarations of interest, these have a transparency objective. The example of D Laurier is used by Tsuda et al. [1] as an argument suggesting a “*failing to disclose a conflicting interest*”. In the reports of international agencies such as IARC or WHO, the criteria for declaration are often explicitly extended to institutional funding sources. This is why it is stated in the IARC report [5] “*Dr Dominique Laurier reports that his institution, Institute for Radiological Protection and Nuclear Safety [IRSN], benefits from research funding from Areva and EDF [French nuclear operators]*”. Such funding is not surprising for a national research and expert body such as IRSN, which has had a charter of ethics and deontology since 2013. In the publication by Cléro et al. in the journal “Environment International” [2], only potential conflicts and individual funding sources are considered. That is why the authors of the article, including D Laurier, stated that “*The authors declare that they have no known competing financial interests or personal relationships that could have appeared to influence the work reported in this paper*”. We maintain there is no “*failing to disclose a conflicting interest*” in the SHAMISEN project.

Finally, Tsuda et al. [1] suggest that the authors of the SHAMISEN paper [2] ignore the difference between a cohort and an ecological epidemiological design. We would like to remind the definition of an ecological study, which is an observational study defined by the level at which the data is analyzed, i.e. at the population or group level, rather than the individual level (see for example [6] for definition). Clearly, the analysis published by Tsuda et al. in 2016 [7] is of ecological nature as also highlighted by Jorgensen [8], and not a cohort study as Tsuda et al. believe.

## Conclusion

In conclusion, we disagree with some of the arguments and criticisms mentioned by Tsuda et al. [1]. We strongly confirm the conclusions and the recommendations derived from the European project SHAMISEN [2, 3].

## Abbreviations

Areva: French nuclear operator conducting activities related to the uranium fuel cycle (today Orano, formerly Cogema then Areva)
EDF: Electricité De France, French nuclear operator producing and supplying electricity
IARC: International Agency for Research on Cancer
IRSN: Institute for Radiological Protection and Nuclear Safety
SHAMISEN: Nuclear Emergency Situations - Improvement of Medical And Health Surveillance
WHO: World Health Organization.
